# Priority Setting: Right Answer to a Far Too Narrow Question? Comment on "Global Developments in Priority Setting in Health"

**DOI:** 10.15171/ijhpm.2017.66

**Published:** 2017-05-30

**Authors:** Ted Schrecker

**Affiliations:** School of Medicine, Pharmacy and Health, Durham University, University Boulevard, Stockton-on-Tees, UK.

**Keywords:** Resource Allocation, Scarcity, Priority-Setting, Neoliberalism, Distributive Justice

## Abstract

In their recent editorial, Baltussen and colleagues provide a concise summary of the prevailing discourse on priority-setting in health policy. Their perspective is entirely consistent with current practice, yet they unintentionally demonstrate the narrowness and moral precariousness of that discourse and practice. I respond with demonstrations of the importance of ‘interrogating scarcity’ in a variety of contexts.

In their recent editorial, Baltussen and colleagues^1^ provide a concise summary of the prevailing discourse on priority-setting in health policy. Their perspective is entirely consistent with current practice, yet they unintentionally demonstrate the narrowness and moral precariousness of that discourse and practice. In the high-income country context, they question neither the stratospheric pricing of new drugs nor the inevitability of fiscal austerity. In the context of lower-income countries, they address neither the unequal distributions of political resources that work against mobilising resources for health services domestically, nor the historical contribution to resource shortages of the large, rich countries that at least until very recently occupied the commanding heights of the world economy – and continue to exercise influence far outside their borders through multilateral institutions like the World Bank and the International Monetary Fund.

Each of these omissions merits brief elaboration. The drug prices they correctly cite as cause for concern^2,3^ are consequences of a dysfunctional regime for financing health innovation that rewards an industry with a long history of corporate criminality^4,5^ by providing expansive patent protection and unquestioning deference to the rights of owners of ‘intellectual property’ – a regime that is of relatively recent origin,^6^ albeit now imposed on much of the world through a process in which “in effect, twelve corporations made public law for the world”^7^ in the form of the TRIPS agreement. Fiscal austerity is, as has once again been pointed out in the United Kingdom, a political choice^8^ – one made in the larger context of a project of shrinking state expenditure to pre-World War II levels,^9,10^ even as the United Kingdom remains near the low end of the high-income country spectrum in terms of the share of its gross domestic product (GDP) devoted to public spending on healthcare. Crucially, at the macro-level of public finance, other jurisdictions are making different choices (Figure 1).

**Figure 1:**
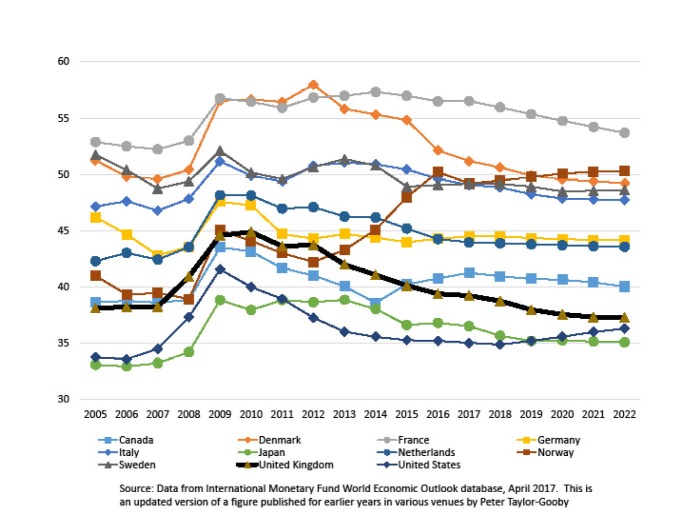
General Government Spending as % of GDP, Selected Countries, Actual and Anticipated.
Source: Data from International Monetary Fund World Economic Outlook database, April 2017. This is an updated version of a figure published for earlier years in various venues by Peter Taylor-Gooby.

Outside the high-income world, recent Chatham House work^11^ suggests that many countries could provide at least basic healthcare for all their residents through more effective mobilisation of domestic resources – a point Reeves and colleagues^12^ have made with specific reference to India, an under-performer on comparative health indicators that at the national level has embarked on a course of cutting public spending on health.^13^ It is the resistance of the privileged to even modest domestic redistribution that often stands in the way and creates the context for priority-setting. International influences are also implicated. The 2014 Ebola outbreak temporarily redirected attention to the debilitating effects on national health systems of decades of ‘structural adjustment’ nostrums promoted by the International Monetary Fund and the World Bank,^14-16^ recently using different labels, in an effort to protect creditor interests and diffuse neoliberal macroeconomic policies that have been widely discredited. Although some of the magnitudes remain contested,^17^ the high-income world is further implicated in facilitating capital flight and tax avoidance mechanisms that reduce the resources available to provide basic healthcare and related needs in many jurisdictions.^18-20^ And the intractable problem of massively inadequate research on diseases of the poor, who do not constitute an attractive market for the pharmaceutical industry,^21,22^ implies the need for urgent attention to priority-setting in quite a different domain.

The usual axiom in priority-setting using cost-effectiveness analysis is that no matter how high the health budget is, it will never be sufficient to fulfil all demands and therefore priority setting will always be needed to ensure resources are not wasted on interventions that do not buy much health. The axiom rests on a missing middle premise: that the current budget is the appropriate one. This premise must be interrogated. The presumption that the fiscal constraint imposed by the overall healthcare budget should be taken as given, and subjected neither to ethical nor political analysis, is questionable even under conditions of formal democracy, which are far from universal. That said, improving mechanisms for priority-setting is important, as is defending the integrity of highly regarded processes like those of the National Institute for Clinical and Health Excellence (NICE) in England^23^ – not least because these serve as a defence against the overblown claims of the pharmaceutical industry. However, better priority-setting that takes macro-level resource constraints as given should not be the primary concern of health systems researchers. In setting priorities for their own work they could do more good, or at least less harm, by ‘interrogating scarcity’^24^: directing their attention and that of their audiences to the political choices, made domestically and internationally, that mean resources are scarce in some settings, and for some purposes, but not in and for others.

In the UK context, various forms of covert and not-so-covert rationing^25,26^ will probably become routine, if that has not already happened. A 2015 Nuffield Trust briefing warned flatly that “the sum of all NICE commissioning guidance for an area would almost certainly be unaffordable”^26^; mainstream academic discussion of the future of the NHS now accepts “the inevitability of hard choices in healthcare.”^27^ Such acceptances come with little scrutiny of the disabling^28^ if not homicidal^29^ impact of those choices, critique of the political commitments driving them, or acknowledgement that they seldom affect the rich. (The Bank of England now advertises that its Deputy Governors’ compensation package includes private medical insurance; see Figure 2).

**Figure 2:**
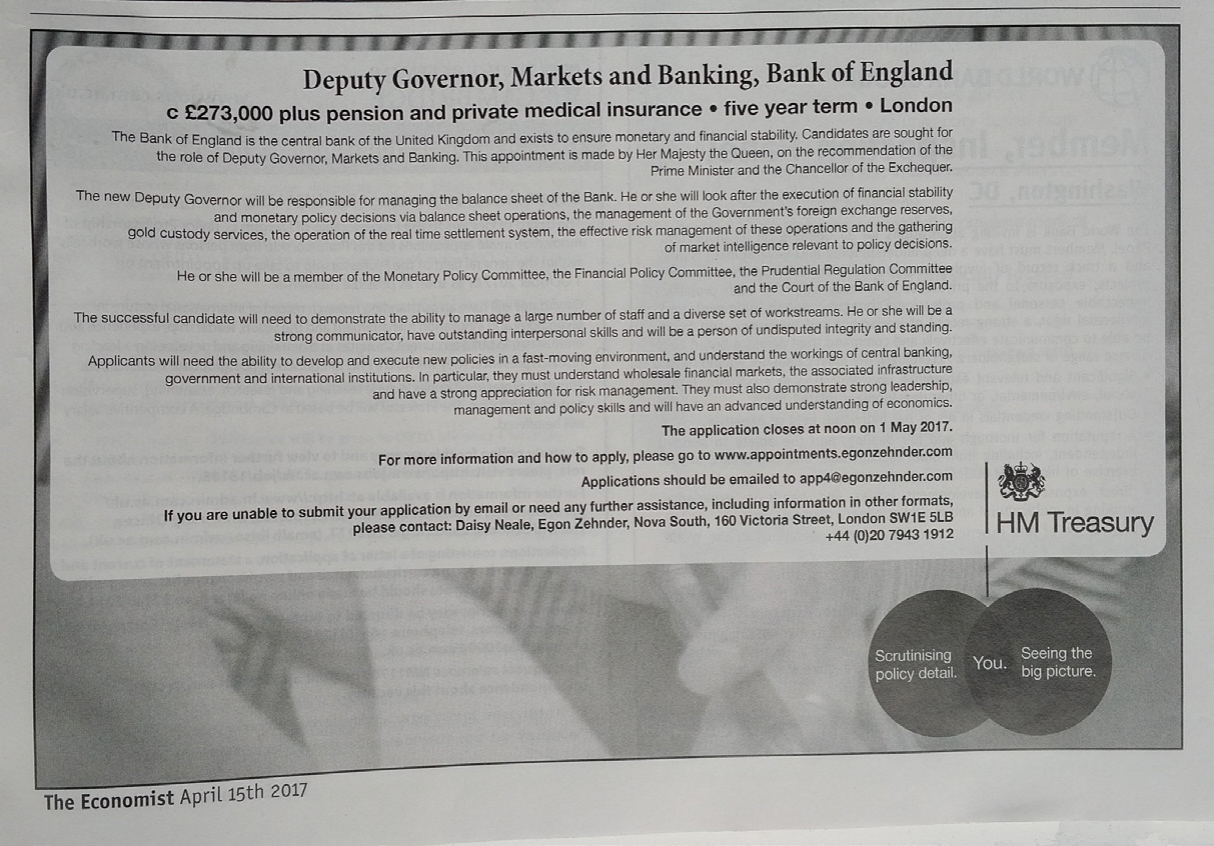
Public Health Cover in the United Kingdom: Not Good Enough for Some.

At the Birmingham conference referred to in the editorial, many participants were preoccupied with how to defend ‘de-commissioning’ of NHS services – obfuscatory jargon for making them available only to those with deep pockets. The conference also featured a session on “Understanding how neoliberalism threatens health, and how to fight back” (full disclosure: organised by the author). One can only wish the editorial’s authors had attended.

## Ethical issues

Not applicable.

## Competing interests

Author declares that he has no competing interests.

## Author’s contribution

TS is the single author of the paper.
